# Recurrent cytokine release syndrome in patients receiving bispecific antibody therapy for multiple myeloma: a case series

**DOI:** 10.1007/s00277-026-06966-6

**Published:** 2026-03-26

**Authors:** Roberta Della Pepa, Aldo Leone, Francesco Grimaldi, Simona Avilia, Mara Memoli, Bernardo Rossini, Carmine Liberatore, Fabrizio Pane

**Affiliations:** 1 Department of Clinical Medicine and Surgery, University Federico II, Naples, Italy; 2 Hematology and Cell Therapy Unit, IRCCS Istituto Tumori ″Giovanni Paolo II″ Bari, Bari, 70124 Italy; 3 Hematology Unit, Department of Oncology and Hematology, Ospedale Santo Spirito, Pescara, Italy; 4 Department of Medicine and Aging Sciences, University of Chieti-Pescara, Chieti, Italy

**Keywords:** Multiple myeloma, bispecific antibody, cytokine release syndrome, immunotherapy, adverse events

## Abstract

Bispecific antibodies targeting B-cell maturation antigen, BCMA (teclistamab) or G protein–coupled receptor, class C, group 5 member D, GPRC5D (talquetamab) are effective treatments for relapsed or refractory multiple myeloma. Their main early toxicity is cytokine release syndrome (CRS), usually transient and limited to initial cycles. We report five patients with relapsed or refractory multiple myeloma who received bispecific antibodies (n = 1 teclistamab; n = 4 talquetamab) who developed a pattern of recurrent, cyclic CRS beyond the initial step-up phase. All patients achieved at least a partial hematologic response, with complete or stringent complete remission in most cases. Recurrent febrile episodes occurred at regular intervals after drug administration, despite repeatedly negative infectious workups. CRS episodes were generally grade 1–2 and resolved with tocilizumab, with or without corticosteroids. In one patient treated with teclistamab, recurrent CRS contributed to treatment discontinuation due to renal complications. Attenuation of CRS severity over time was observed in some patients, allowing treatment continuation with tailored premedication strategies. This case series describes an unusual pattern of recurrent CRS occurring during bispecific antibody therapy for multiple myeloma. Recognition of this phenomenon is essential to avoid misdiagnosis as infection and unnecessary antimicrobial use. Systematic reporting of such real-world adverse events may help refine supportive care strategies.

## Introduction

Bispecific T-cell engagers (BiTEs) targeting BCMA (e.g., teclistamab) or GPRC5D (e.g., talquetamab) have emerged as promising therapeutic options for relapsed/refractory multiple myeloma (RRMM) patients, offering deep and durable responses in heavily pretreated populations [1, 4]. Both agents are associated with high rates of cytokine release syndrome (CRS), generally occurring during the initial step-updosing and resolving with supportive care [2, 5].

Recurrent CRS beyond early treatment cycles has been only sporadically described. Here, we report five patients who experienced repeatedly occurring CRS episodes during ongoing bispecific antibody therapy, highlighting diagnostic challenges and management strategies.

## Case Descriptions

### Case 1

A 64-year-old woman with IgG kappa MM (ISS I) diagnosed in 2019 received multiple treatment lines, including Bortezomib, Thalidomide, Dehamethasone (VTD) induction (discontinued due to severe COVID-19 infection). At progression, she was treated with four cycles of daratumumab–lenalidomide–dexamethasone (DRd) followed by ASCT, then continued DRd until cycle 12, when she relapsed with multiple skeletal lesions (SUVmax up to 35 on PET-CT). She subsequently received eight cycles of carfilzomib–dexamethasone, after which disease progression occurred.

In July 2023, teclistamab was initiated under a compassionate-use program, achieving complete biochemical response. During step-updosing, administered per protocol with standard premedication (steroid, antihistamine, and antipyretic) she developed grade 2 CRS (day + 2), managed with tocilizumab. During outpatient therapy, recurrent febrile episodes (up to 39 °C) occurred consistently 48 h post-infusion and were managed with corticosteroids and paracetamol. Extensive infectious evaluations (blood/urine cultures, chest imaging, echocardiography) were repeatedly negative.

After 12 months, she maintained complete response, though two residual paraosseous lesions requie local radiotherapy. During maintenance teclistamab dosing, she experienced an intense grade 3 CRS episode, successfully managed with corticosteroids and tocilizumab. Laboratory tests persistently showed elevated inflammatory markers (C-Reactive Protein, Erythrocyte Sedimentation Rate), leukocytosis, and thrombocytosis, despite negative infectious workups.

In January 2025, renal function began to deteriorate, leading to discontinuation of teclistamab in March 2025. Renal biopsy revealed interstitial nephritis unrelated to MM. She was started on prednisone 1 mg/kg/day with initial improvement in renal function. At last follow-up, she remained in stringent complete response, on steroid tapering, though inflammatory markers remained elevated three months after teclistamab discontinuation.

### Case 2

A 65-year-old man with IgG kappa Multiple myeloma (MM) (Durie-Salmon IIIB) diagnosed in 2012 underwent multiple lines of therapy, including Autologous stem celltransplant (ASCT) and proteasome inhibitor (PI)/Immunomodulatory agents (IMiDs)/anti-CD38 combinations. Following multiple relapses, he started talquetamab in March 2023 within a compassionate-use program, achieving complete hematologic response.

During step-updosing, he developed, despite premedication (steroid, antihistamine, and antipyretic), grade 2 CRS on day + 2 post-first dose, managed with paracetamol and tocilizumab, resolving within 24 h. After transition to biweekly outpatient infusions, recurrent febrile episodes (up to 39 °C) occorre consistently 24–48 h post-administration every 35–40 days.

An extensive infectious workup was performer at each episode: blood cultures, urinalysis, chest imaging [X-ray and Computed Tomography (CT)], CMV PCR, COVID-19 RT-PCR, and echocardiography—all negative. No bacterial, viral, or fungal infections were detected.

Management included administration of tocilizumab (8 mg/kg) with complete symptom resolution, alongside prophylactic dexamethasone (16 mg/day, tapered over three days post-infusion).

### Case 3

A 71-year-old woman with IgG lambda MM (Durie-Salmon IIIA) was heavily pretreated across multiple lines, including ASCT, PIs, IMiDs, daratumumab, and belantamab mafodotin. In January 2024, she initiated talquetamab under a compassionate-use protocol.

During step-up dosing (administered after standard premedication), she developed grade 2 CRS (day + 3 post-first dose), requiring tocilizumab. Subsequently, recurrent fever episodes (every 30–35 days) consistently followed talquetamab infusions.

Infectious screening included blood/urine cultures, nasal/throat/rectal swabs, CMV PCR, COVID-19 testing, beta-D-glucan, galactomannan, and chest CT scans; no infectious source was identified.

Management consisted of initial corticosteroids (prednisone 25 mg/day), ineffective alone, followed by tocilizumab with rapid resolution. Recurrent CRS was managed with repetitive tocilizumab use. Additional adverse events included palmar desquamative dermatitis and onychopathy, treated with topical tacrolimus, and transient ageusia.

### Case 4

A 75-year-old woman with non-secretory IgA lambda MM (Durie-Salmon IIIA) and extensive prior therapy, including ASCT, radiotherapy, PIs, IMiDs, daratumumab, and belantamab mafodotin, started talquetamab after disease progression.

During step-updosing, she developed grade 2 CRS despite premedication (day + 2 post-first dose), requiring tocilizumab. Regardless of successful initial management, she experienced recurrent febrile episodes monthly.

Infectious workups—including blood cultures, chest CT scans, and COVID-19 testing—identified two documented infections: COVID-19 pneumonia in December 2023 and bronchitis in January 2025, the latter managed with empirical broad-spectrum antibiotics. However, several febrile episodes lacked a clear infectious source and were attributed to CRS. These episodes consistently responded to tocilizumab administration.

### Case 5

A 69-year-old woman with light-chain kappa MM (ISS II, R-ISS II) diagnosed in 2018 underwent multiple treatment lines, including VTD induction, single ASCT with lenalidomide maintenance, followed, at relapse, by pomalidomide, bortezomib–dexamethasone, and daratumumab–bortezomib–dexamethasone. In October 2023, she relapsed with symptomatic disease and initiated talquetamab within a compassionate-use program, being triple-class refractory.

Step-up dosing (administered inpatient and premedicated with paracetamol, cetirizine and dexamethasone) was well tolerated. At 1 month, she achieved an early and deep response (serum and urine immunofixations negative). Shortly thereafter, she developed right oculomotor nerve palsy, managed with intravenous immune globulin (IVIG) administration and prednisone 1 mg/kg, with gradual clinical recovery. Steroids were tapered, and talquetamab was continued every two weeks.

Two months after treatment initiation, she developed grade 1 oral- and skin-related adverse events (xerostomia, dysgeusia, onychodystrophy) and a paucisymptomatic SARS-CoV-2 infection, requiring temporary discontinuation of therapy. After recovery, talquetamab was resumed biweekly with monthly IVIG supplementation for hypogammaglobulinemia.

At 6 months, she experienced fever and diarrhea due to *Salmonella enteritidis* infection, successfully treated with ceftriaxone. After recovery, talquetamab was restarted every two weeks, with stringent CR documented at 9 months. Despite diarrhea resolution, she developed recurrent low-grade fevers, typically occurring soon after infusions.

Extensive infectious workups (blood/urine/stool cultures, chest CT, abdominal ultrasound, echocardiography, CMV/EBV/HSV-1 and − 2/HHV-6 PCR, SARS-CoV-2 and respiratory viral swabs) were consistently negative. Fevers persisted despite monthly IVIG and multiple courses of empirical antibiotics, and were attributed to late-onset grade 1 CRS.

Initial management with on-demand prednisone 25 mg/day achieved only partial control. Considering the deep response and concomitant grade 2 weight loss secondary to oral off-target toxicities, talquetamab was reduced to monthly administration, resulting in complete CRS resolution.

At 18 months from talquetamab initiation, the patient is in good clinical condition, afebrile, and in persistent stringent CR.

The main clinical characteristics, timing of CRS onset, management strategies, and impact on treatment for all five patients are summarized in Table 1.

**Table 1:** Summary of patients with recurrent CRS during bispecific antibody therapy

Case	Age/Sex	MM subtype	Prior lines of therapy	Bispecific antibody	First CRS (timing, grade)	Recurrent CRS (timing)	Premedication	CRS management	Impact on treatment	Learning points
1	64/F	IgG κ	4	Teclistamab	C1 D2, G2	Every 7 days, 48 h post-infusion	Paracetamol Cetirizine Dexamethasone	Tocilizumab ± steroids	Discontinued (renal toxicity)	Recurrent CRS may contribute to treatment discontinuation
2	65/M	IgG κ	4	Talquetamab	C1 D2, G2	Every 35–40 days, 24–48 h post-infusion	Paracetamol Cetirizine Dexamethasone	Tocilizumab ± steroids	Continued	Predictable cyclic CRS responsive to IL-6 blockade
3	71/F	IgG λ	5	Talquetamab	C1 D3, G2	Every 30–35 days post-infusion	Paracetamol Cetirizine Dexamethasone	Tocilizumab ± steroids	Continued	Tocilizumab required despite steroid premedication
4	75/F	IgA λ	5	Talquetamab	C1 D2, G2	Every 30 days post-infusion	Paracetamol Cetirizine Dexamethasone	Tocilizumab	Continued	CRS may coexist with true infection
5	69/F	light-chain κ	4	Talquetamab	None during step-up dose	Every 15 days, 24 h post-infusion	Paracetamol Cetirizine Dexamethasone	Steroids	BsAb dose reduced to monthly administration	Dose adaptation may resolve recurrent CRS

## Discussion

CRS is a common and well-characterized immune-related adverse event associated with bispecific antibody therapy, typically occurring during early treatment cycles as a consequence of acute T-cell activation [1–3]. In pivotal trials, CRS incidence reached approximately 77% with talquetamab in MonumenTAL-1 [1] and about 70% with teclistamab in MajesTEC-1 [4, 5], with most events being grade 1–2 and confined to step-updosing. Standard management includes antipyretics, corticosteroids, and IL-6 receptor blockade with tocilizumab.

In contrast, our series of patients treated with teclistamab (*n* = 1) and talquetamab (*n* = 4) describes an unusual pattern of recurrent, cyclic CRS occurring throughout ongoing therapy, independent of initial priming. CRS episodes recurred at predictable intervals after drug administration, consistently resolved with tocilizumab, and were not associated with documented bacterial, viral, or fungal infections, supporting an immune-mediated rather than infectious etiology. Treatment delays and discontinuation occorre primarily in the teclistamab-treated patient, whereas most talquetamab-treated patients were able to continue therapy with schedule adaptation. Importantly, CRS management did not appear to compromise anti-myeloma efficacy, in line with prior observations in bispecific-treated cohorts [4, 5].

Several mechanisms maycontribute to this atypical presentation, including sustained low-level T-cell engagement and impaired resolution of inflammatory responses in heavily pretreated patients [6–8]. Emerging translational data suggest that chronic bispecific stimulation can maintain intermittent cytokine production even in the absence of high tumor burden [9, 10]. This phenomenon may be particularly relevant for GPRC5D-targeting agents such as talquetamab, given low-level antigen expression in non-malignant epithelial tissues [11].

Advanced age, extensive prior therapies, and immune dysregulation may further predispose patients to repetitive IL-6–driven inflammatory responses, explaining the recurrent nature of CRS observed in our cohort and its consistent responsiveness to IL-6 blockade [12, 13].

Clinically, recurrent CRS did not correlate with disease progression or depth of response, highlighting the importance of avoiding misinterpretation of fever as a surrogate marker of treatment efficacy. Recognition of late or recurrent CRS is crucial to prevent unnecessary antimicrobial use and to guide appropriate supportive care. However, our observations are based on a limited cohort of five patients and should therefore be interpreted with caution. For this reason, our findings underscore the need for aggregation of similar cases across treating centers to better define the incidence, clinical relevance, and optimal management of recurrent CRS associated with bispecific antibodies, ultimately improving treatment safety and patient management.

## Conclusions

Recurrent CRS during ongoing bispecific antibody therapy represents a clinically relevant but potentially underrecognized toxicity that warrants increate awareness in routine practice.

## Data Availability

The data that support the findings of this study are available from the corresponding author upon reasonable request. All data will be provided in an anonymized form to protect patient privacy. Data are stored in a controlled access repository at the University of Federico II in Naples.
